# Risk factors for the recurrence of cervical cancer using MR-based T1 mapping: A pilot study

**DOI:** 10.3389/fonc.2023.1133709

**Published:** 2023-03-16

**Authors:** Jie Liu, Shujian Li, Qinchen Cao, Yong Zhang, Marcel Dominik Nickel, Yanglei Wu, Jinxia Zhu, Jingliang Cheng

**Affiliations:** ^1^ Department of Magnetic Resonance, The First Affiliated Hospital of Zhengzhou University, Zhengzhou, China; ^2^ Department of Radiotreatment, The First Affiliated Hospital of Zhengzhou University, Zhengzhou, China; ^3^ Magnetic Resonance (MR) Application Predevelopment, Siemens Healthcare Gesellschaft mit beschrankter Haftung (GmbH), Erlangen, Germany; ^4^ Magnetic Resonance (MR) Collaboration, Siemens Healthineers Ltd., Beijing, China

**Keywords:** cervical cancer, magnetic resonance imaging, native T1, recurrence, risk factors

## Abstract

**Objectives:**

This study aimed to identify risk factors for recurrence in patients with cervical cancer (CC) through quantitative T1 mapping.

**Methods:**

A cohort of 107 patients histopathologically diagnosed with CC at our institution between May 2018 and April 2021 was categorized into surgical and non-surgical groups. Patients in each group were further divided into recurrence and non-recurrence subgroups depending on whether they showed recurrence or metastasis within 3 years of treatment. The longitudinal relaxation time (native T1) and apparent diffusion coefficient (ADC) value of the tumor were calculated. The differences between native T1 and ADC values of the recurrence and non-recurrence subgroups were analyzed, and receiver operating characteristic (ROC) curves were drawn for parameters with statistical differences. Logistic regression was performed for analysis of significant factors affecting CC recurrence. Recurrence-free survival rates were estimated by Kaplan–Meier analysis and compared using the log-rank test.

**Results:**

Thirteen and 10 patients in the surgical and non-surgical groups, respectively, showed recurrence after treatment. There were significant differences in native T1 values between the recurrence and non-recurrence subgroups in the surgical and non-surgical groups (P<0.05); however, there was no difference in ADC values (P>0.05). The areas under the ROC curve of native T1 values for discriminating recurrence of CC after surgical and non-surgical treatment were 0.742 and 0.780, respectively. Logistic regression analysis indicated that native T1 values were risk factors for tumor recurrence in the surgical and non-surgical groups (P=0.004 and 0.040, respectively). Compared with cut-offs, recurrence-free survival curves of patients with higher native T1 values of the two groups were significantly different from those with lower ones (P=0.000 and 0.016, respectively).

**Conclusion:**

Quantitative T1 mapping could help identify CC patients with a high risk of recurrence, supplementing information on tumor prognosis other than clinicopathological features and providing the basis for individualized treatment and follow-up schemes.

## Introduction

Cervical cancer (CC) is the second leading gynecological malignancy that seriously affects women’s health worldwide (1). According to a 2018 epidemiological investigation, approximately 570,000 women have CC every year, with a mortality rate of 54.6% (2). The International Federation of Gynecology and Obstetrics (FIGO) staging provides the basis for optimal CC treatment at the time of diagnosis. Different treatment options are recommended for each CC stage. Surgery is the first-line therapy for early-stage CC, whereas concurrent chemoradiotherapy (CCRT) is the primary treatment for advanced CC (3). A previous study showed that the 5-year recurrence rate of CC is approximately 28% (4), ranging from 10–20% for patients who underwent surgery (5, 6) and approximately 32% for patients who did not undergo surgery (7). In addition to the differentiation and heterogeneity of the tumor, recurrence is also related to inaccurate staging and insufficient evaluation in peripheral invasion (8). The current clinical options for further treatment of recurrent lesions are limited, with the one-year survival rate ranging from 15–20% (9, 10). Timely identification of patients with a high risk of recurrence will aid the development of individualized treatment and follow-up plans (11, 12). Previous studies suggested that most risk factors, such as tumor size, lymph node metastasis, and parametrial invasion, could only be accurately evaluated based on postoperative pathologic examination and are, therefore, of limited value in guiding therapeutic decisions. Thus, it is necessary to seek reliable biomarkers to improve the capability of identifying patients with a high risk of recurrence before treatment.

Medical imaging is of critical clinical importance in the diagnosis and prediction of cancer prognosis (7). Of the numerous imaging methods used for examining patients with cancer, magnetic resonance imaging (MRI) is the best for evaluating the pathologic features and prognostic factors of CC owing to its high soft tissue resolution, safety, and diverse imaging modes and parameters (13, 14). Several relevant studies based on functional MRI have been conducted to predict the prognosis of CC after definitive therapy. For example, in one retrospective study of 31 patients with CC treated with radiation therapy, the pre-treatment ADC mean values for primary CC tumors with recurrence were lower than those without recurrence (15). However, Heo et al. demonstrated that the pre-treatment ADC_mean_ values of CC tumors were significantly higher in the recurrence group than in the non-recurrence group (16). Apart from the number and heterogeneity of the patient population and retrospective study design, the conflicting results of these previous studies may also be attributed to different MRI imaging protocols and non-standardized parameter settings.

T1 mapping is a quantitative MRI diagnosis technology that is independent from technical implications (17). Two techniques are used to acquire T1 maps; inversion recovery and saturation recovery (18). The former technique is more widely used in clinical practice because of its demonstrated high accuracy (19). The Look-Locker inversion recovery sequence is among the most efficient methods for T1 measurement as it can capture multiple images after each inversion pulse (20). The T1 value, also known as the longitudinal relaxation time, is the decay constant for the exponential recovery of the longitudinal magnetization toward its equilibrium state (21). Quantitative T1 mapping can directly reflect the microscopic alterations and potential pathophysiologic processes in tissues by measuring their T1 value (22). In the early stages of most diseases, tissues show biochemical changes and increases in water content (23, 24). Thus, T1 relaxation time, mainly determined based on interstitial tissue water (25), has been recommended as a biomarker for early diagnosis of diseases (26). Lescher et al. (27) and Qin et al. (11) found that T1 mapping helps monitor tumor progression and prognosis in patients with glioblastoma and hepatocellular carcinoma, respectively. Other studies have confirmed that T1 mapping is beneficial for assessing the histopathological features of tumors and diagnosing tumor recurrence (28, 29). However, although T1 mapping is increasingly used in tumor studies (30, 31), the impact of utilizing T1 values in assessing the prognosis of patients with CC has not been investigated.

In this study, we used conventional diffusion-weighted imaging (DWI) as a reference to explore whether post-treatment recurrence of patients with CC can be reflected in MRI-based T1 mapping.

## Materials and methods

### Patient population

**Figure 1:**
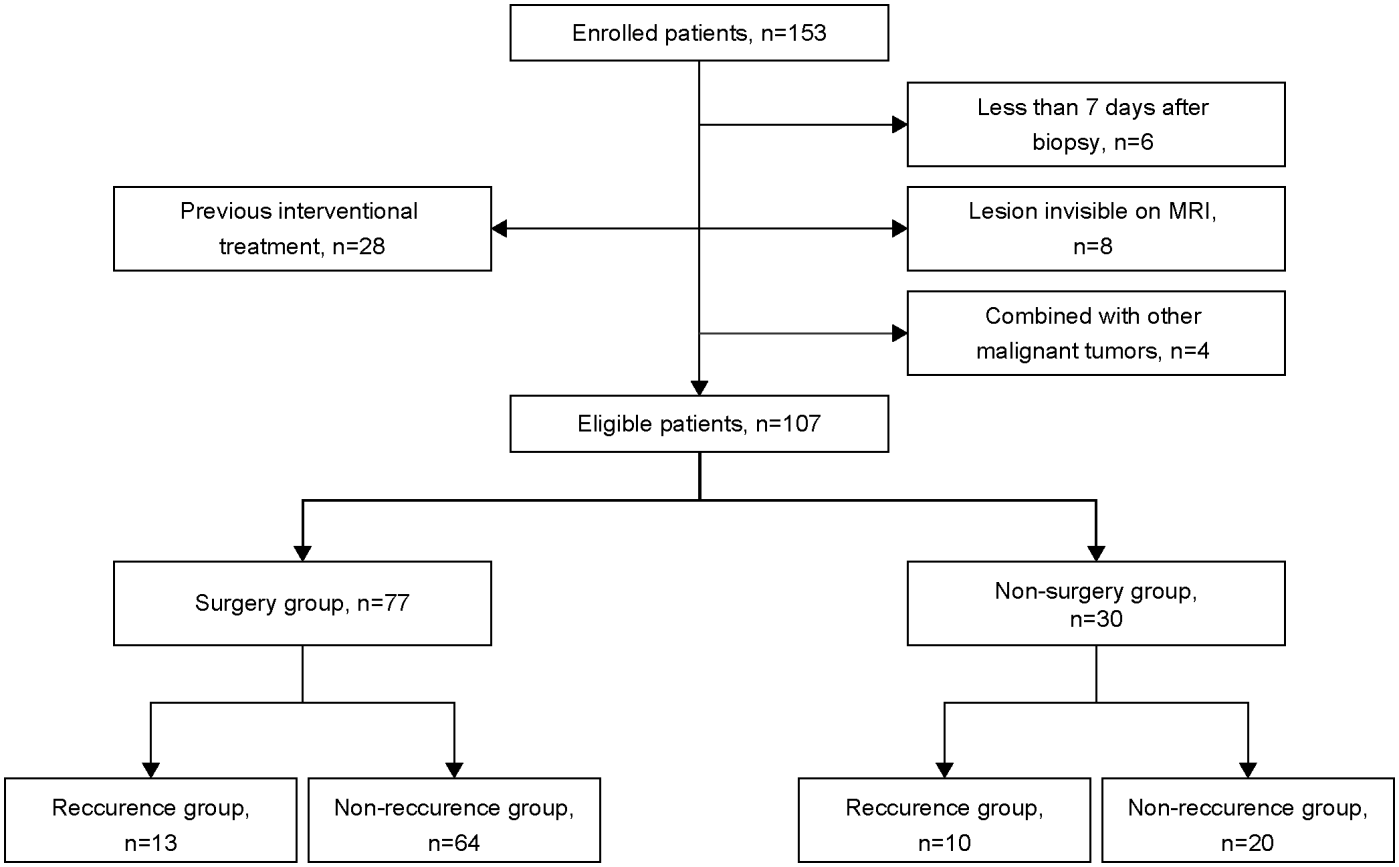
Flowchart of patient enrolment.

A total of 153 patients who were histopathologically diagnosed with CC between May 2018 and April 2021 were enrolled in this prospective study. The inclusion criteria for this study were as follows: i) patients diagnosed with CC based on the results of histopathological staining, ii) patients who underwent surgery or standard CCRT within 1 month after MRI examination, and iii) patients with tumors ≥1 cm in size. The exclusion criteria were as follows: i) patients who underwent MRI less than 7 days after the cervical biopsy, ii) patients who received previous interventional treatment, and iii) patients with a history of other malignant tumors. The final study cohort consisted of 107 patients with CC (**Table 1**, **Figure 1**). The tumor types and degrees of pathological differentiation were classified based on the World Health Organization classification.

**Table 1:** Summary of patient data.

Characteristics	Surgical group (n=77)		Non-surgical group (n=30)	
	Recurrence (n=13)	Non-recurrence (n=64)	Recurrence (n=10)	Non-recurrence (n=20)
Mean age, years (range)	51.85 (25–67)	52.02 (29–73)	52.70 (39–71)	52.15 (32–48)
FIGO stage, n (%)				
IB	5 (38.5)	20 (31.2)	NA	NA
IIA	8 (61.5)	44 (68.8)	NA	NA
IIB	NA	NA	3 (30.0)	7 (35.0)
III	NA	NA	3 (30.0)	9 (45.0)
IV	NA	NA	4 (40.0)	4 (20.0)
Histology type, n (%)				
SCC	10 (76.9)	54 (84.4)	8 (80.0)	19 (95.0)
Non-SCC	3 (23.1)	10 (15.6)	2 (20.0)	1 (5.0)
Tumor differentiation, n (%)				
Poorly differentiated	3 (23.1)	14 (21.9)	3 (30.0)	3 (15.0)
Moderately differentiated	6 (46.1)	34 (53.1)	1 (10.0)	5 (25.0)
Well-differentiated	NA	1 (1.6)	NA	NA
Unclear	4 (30.8)	15 (23.4)	6 (60.0)	12 (60.0)
Maximum tumor size, n (%)				
<4 cm	5 (38.5)	34 (53.1)	3 (30.0)	12 (60.0)
≥4 cm	8 (61.5)	30 (46.9)	7 (70.0)	8 (40.0)
Lymph node invasion, n (%)				
Negative	8 (61.5)	57 (89.1)	6 (60.0)	13 (65.0)
Positive	5 (38.5)	7 (10.9)	4 (40.0)	7 (35.0)
Mean recurrence time, months (range)	14.69 (3–27)	NA	10.10 (2–21)	NA

FIGO, International Federation of Gynecology and Obstetrics; NA, not available; SCC, squamous cell carcinoma.

This study was approved by the Ethics Committee of the First Affiliated Hospital of Zhengzhou University (approval number: 2021-KY-0133-002). Written informed consent was obtained from all the patients before this study's enrolment.

### Treatment

Patients in the surgical group underwent radical hysterectomy and pelvic lymphadenectomy with or without adjuvant therapy. In contrast, those in the non-surgical group received volume-modulated radiotherapy of 45 Gy (total dose) administered at 1.8–2 Gy per session, five times per week, according to the National Comprehensive Cancer Network Guidelines for Cervical Cancer (version 3.2019) (32). The specific irradiation target areas included the CC, parauterine tissue, and some lymph node drainage areas (including the internal, external, common iliac, and obturator lymph node chains). A 30 mg/m^2^ dose of cisplatin-sensitized chemotherapy was administered simultaneously with radiation. Intravenous drips were administered on radiotherapy days 1, 8, 15, 22, and 29. Intracavitary afterloading radiotherapy was administered as well. The total radiotherapy dose was 35 Gy, administered 5–7 Gy per session, 1–2 times per week. The total duration of treatment was 5–6 weeks.

### Follow-up

All patients were clinically and radiologically followed up for 6 months to 3 years, and recurrence (including distant metastasis) and recurrence time were recorded. Recurrence was diagnosed through medical imaging (PET/CT, CT, or MRI) or pathological confirmation. Follow-up was conducted every 3–4 months in the first 2 years and every 6 months in the third year after treatment. The follow-up phase lasted until November 1, 2021.

### Magnetic resonance imaging protocols

All MRI examinations were performed using a 3T MR scanner (MAGNETOM Skyra; SiemensHealthcare, Erlangen, Germany) with an 18-channel body coil and an integrated 32-channel spine matrix coil. Patients were asked to eat nothing for 4–6 hours before the examination but to drink some water to ensure that the bladder was moderately filled. The examination position was the head-first supine position. The image acquisition range was from the upper edge of the bilateral iliac bone wings to the level of the femoral neck. The patients were instructed to keep their bodies motionless and breathing calm during the scanning process. The MRI protocol included T1-weighted imaging (T1WI), T2-weighted imaging (T2WI), T1 mapping, and DWI. The detailed MRI parameters were as follows:

i) Axial TSE T1WI: repetition time (TR) = 450 ms, echo time (TE) = 18 ms, field of view (FOV) = 320 mm × 320 mm, matrix = 448 × 314, slice thickness = 4 mm, and acquisition time (TA) = 1 min 33 s
ii) Axial TSE T2WI: TR = 3000 ms, TE = 116 ms, FOV = 180 mm × 180 mm, matrix = 384 × 269, slice thickness = 4 mm, and TA = 3 min 20 s
iii) Sagittal TSE T2WI: TR = 2200 ms, TE = 90 ms, FOV = 240 mm × 240 mm, matrix = 384 ×257, slice thickness = 5 mm, and TA = 2 min 16 s
iv) Coronal TSE T2WI: TR = 2200 ms, TE = 85 ms, FOV = 240 mm × 240 mm, matrix = 384 × 257, slice thickness = 3 mm, and TA = 2 min 36 s
v) Axial DWI was performed using a single-shot echo-planar imaging protocol: TR = 4400 ms, TE = 85 ms, FOV = 280 mm × 280 mm, matrix = 154 × 131, slice thickness = 5 mm, and TA = 57 s. Diffusion gradients were applied in three directions with two b-values of 0 and 800 s/mm^2^.
vi) T1 mapping was acquired using a prototypic Look-Locker T1 mapping sequence: TR = 3.0 ms, TE = 1.32 ms, FOV = 300 mm × 300 mm, matrix = 128 × 64, slice thickness = 4 mm, and TA = 1 min 32 s. Thirty-two contrasts were acquired after a 180° inversion pulse along the T1 recovery curve.

Both apparent diffusion coefficient (ADC) and T1 maps were automatically generated inline after image acquisition.

### Image data analysis and processing

**Figure 2:**
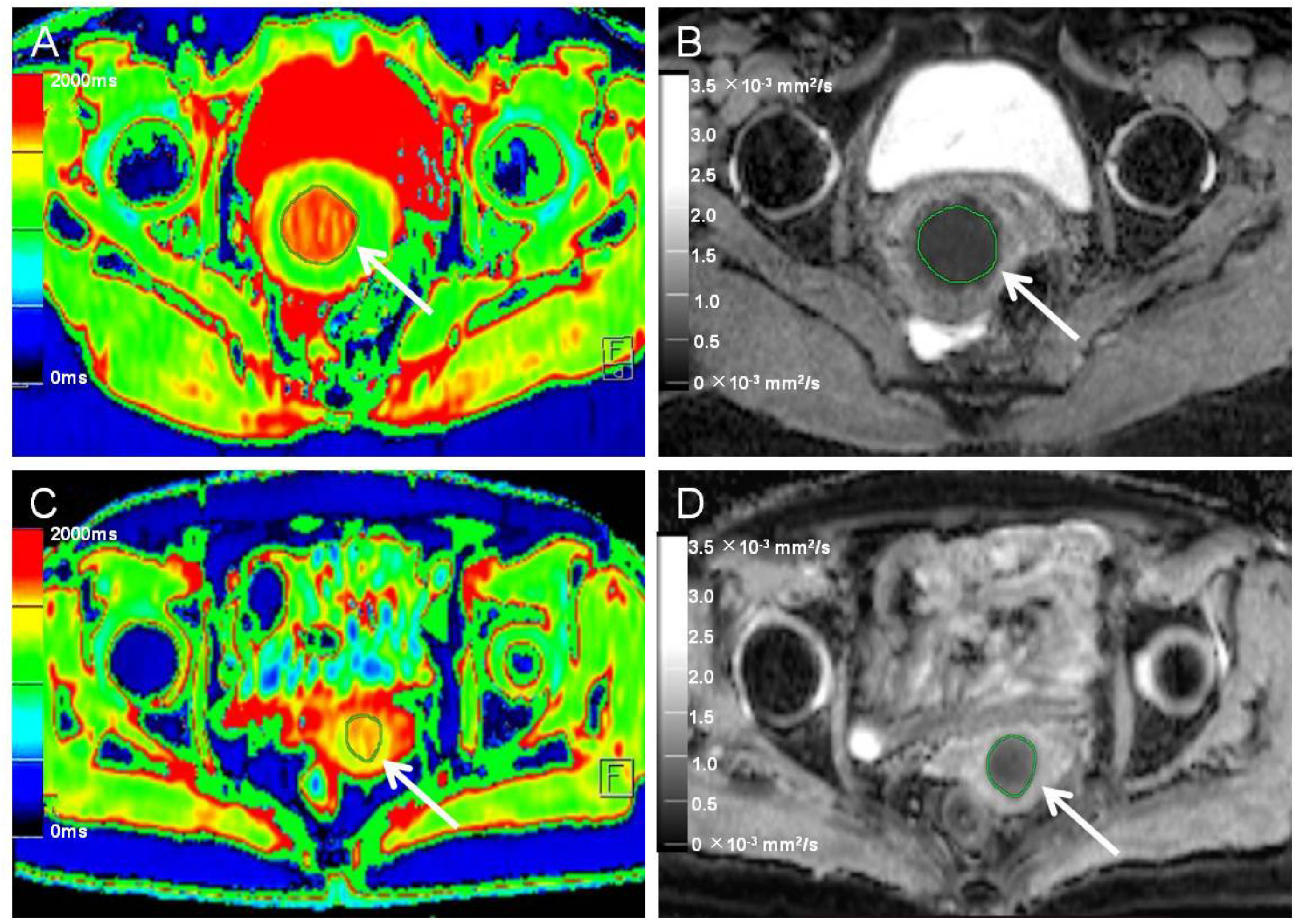
Native T1 mapping and apparent diffusion coefficient (ADC) images of recurrent and nonrecurrent cervical cancer (CC) in the surgical group. **(A, B)** A 45-year-old patient with recurrence during the follow-up period. **(A)** Axial T1 mapping pseudo-color map and **(B)** axial ADC image. The native T1 and ADC values were 1619.20 ms and 0.68 × 10^−3^ mm^2^/s, respectively. **(C, D)** A 61-year-old patient without recurrence during the follow-up period. **(C)** Axial T1 mapping pseudo-color map and **(D)** axial ADC image. The native T1 and ADC values were 1480.19 ms and 0.82 × 10^−3^ mm^2^/s, respectively. The white arrows in A-D indicate the locations of the lesions.

**Figure 3:**
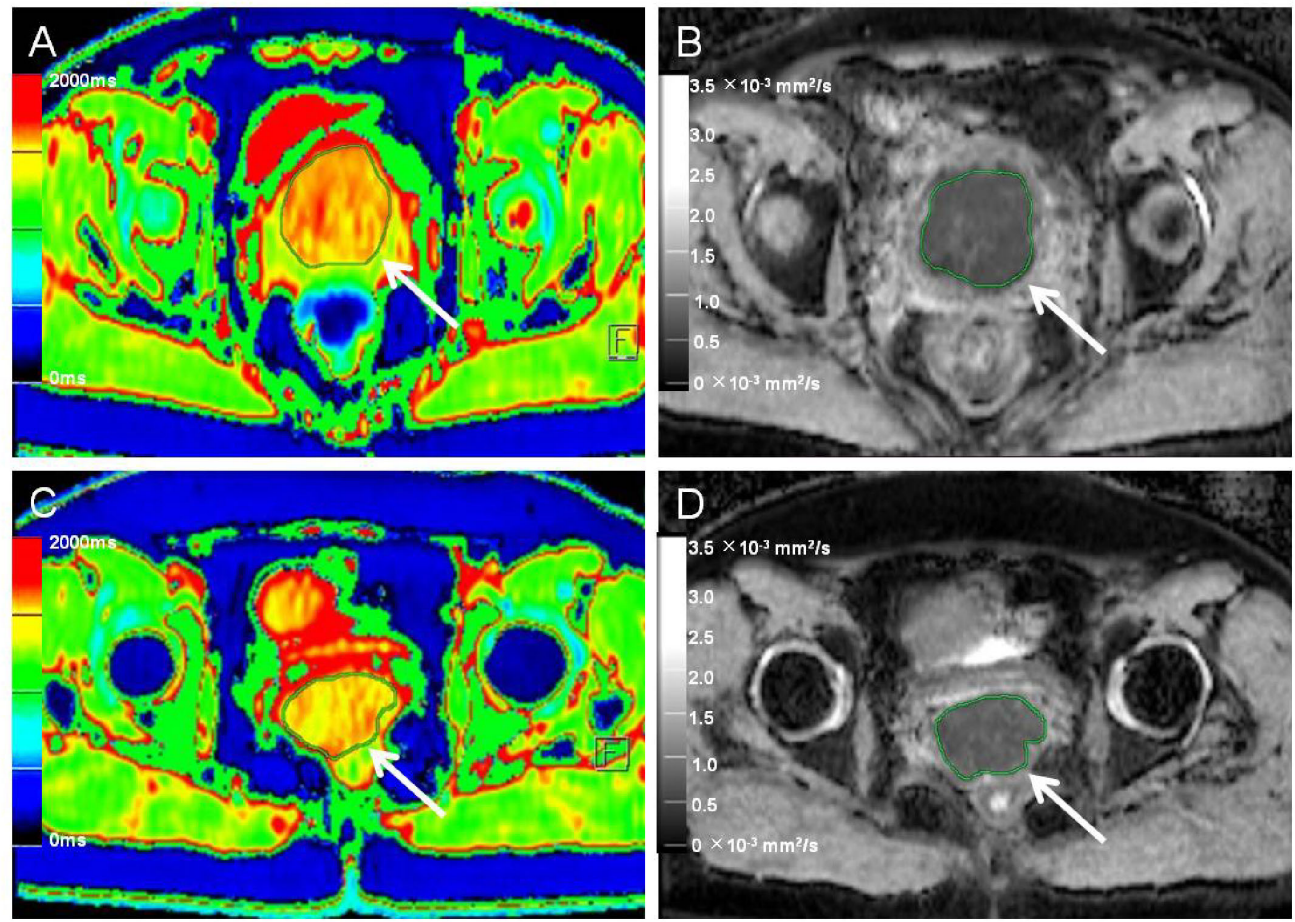
Native T1 mapping and apparent diffusion coefficient (ADC) images of recurrent and nonrecurrent cervical cancer (CC) in the non-surgical group. **(A, B)** A 55-year-old patient with recurrence during the follow-up period. **(A)** Axial T1 mapping pseudo-color map and **(B)** axial ADC image. The native T1 and ADC values were 1556.65 ms and 0.77 × 10^−3^ mm^2^/s, respectively. **(C, D)** A 51-year-old patient without recurrence during the follow-up period. **(C)** Axial T1 mapping pseudo-color map and **(D)** axial ADC image. The native T1 and ADC values were 1489.09 ms and 0.84 × 10^−3^ mm^2^/s, respectively. The white arrows in A–D indicate the locations of the lesions.

The T1 pre-enhancement (native T1) and ADC values of the patients with CC were independently analyzed by two experienced radiologists (with 5 and 10 years of experience in the diagnosis of gynecological tumors) using a post-processing workstation (syngo.via; Siemens Healthcare, Erlangen, Germany). The region of interest (ROI) was manually depicted on the T1 and ADC maps, regarding the conventional T2WI and DWI images, avoiding the cystic or necrotic areas within the lesion (**Figures 2**, **3**). Native T1 and ADC values on all slices of the whole tumor were measured. The average values of these measurements were used for statistical analyses. The maximum tumor diameter was quantitatively evaluated on T2WI. Both radiologists were blinded to the clinicopathological findings. In the surgical group, lymph node metastasis was assessed based on the postoperative pathological findings. In the non-surgical group, any lymph nodes with a short-axis diameter of >10 mm identified on MRI were considered positive indications of metastatic lymph nodes. The diameters were measured using the transverse plane on T2WI, with a slice thickness of 5 mm.

### Statistical analysis

Statistical analyses were performed using SPSS statistical software (version 22; SPSS, Chicago, IL, USA). The differences in clinicopathological variables between recurrence and non-recurrence subgroups in the surgical and non-surgical groups were compared using the Chi-square test. The normality of the distributions of all continuous variables was evaluated using the Kolmogorov–Smirnov test. The normally distributed variables are expressed as mean ± standard deviation. An independent samples t-test was used to compare the differences between the native T1 and ADC values of patients who showed post-treatment recurrence and those who did not. The diagnostic performances of statistically different parameter values were determined using receiver operating characteristic (ROC) curves, which were drawn using the MedCalc V19.0 software (MedCalc Software, Mariakerke, Belgium). The area under the curve (AUC), sensitivity, and specificity were calculated, and the cut-off value for predicting CC recurrence after treatment was obtained using the Youden index. Logistic regression analysis was performed to test the factors that affect the post-treatment recurrence of CC. The Kaplan–Meier method was used to compute the recurrence-free survival rate (RFSR), and the log-rank test was used to compare patient groups. The interobserver variability of the acquired quantitative values was analyzed using the intraclass correlation coefficient (ICC). *P* values <0.05 were considered statistically significant.

## Results

A total of 107 patients (77 in the surgical group and 30 in the non-surgical group) were enrolled in this study. The median age of the patients was 52.1 years (range: 25–73 years). Based on the FIGO system, clinical staging of the tumors revealed that 25 patients had stage Ib cancer, 52 had stage IIa cancer, 10 had stage IIb cancer, 12 had stage III cancer, and eight had stage III-IV cancer. The patients were further categorized into recurrence and non-recurrence subgroups according to their follow-up results. Thirteen patients in the surgical group showed recurrence over 3–27 months of follow-up (average, 14.7 months; recurrence rate, 16.9%). Ten patients in the non-surgical group showed recurrence over 2–21 months of follow-up (average, 10.1 months; recurrence rate, 33.3%) (**Table 1**).

**Figure 4:**
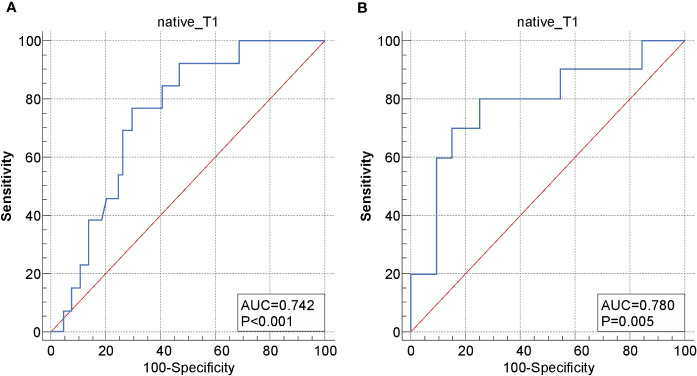
Receiver operating characteristic (ROC) curve of the native T1 values for distinguishing between the recurrence and non-recurrence subgroups in the **(A)** surgical and **(B)** non-surgical groups.

The ICC of the native T1 values (ICC, 0.923; 95% CI, 0.874–0.966), ADC values (ICC, 0.956; 95% CI, 0.916–0.974), and maximum tumor size (ICC, 0.992; 95% CI, 0.988–0.995) showed significant interobserver agreement (33). Lymph node status was significantly different between the recurrence and non-recurrence subgroup in the surgical group (*P*<0.05), while the FIGO stage, histology type, tumor differentiation, and maximum tumor size were not (*P*>0.05) (**Table 2**). Regardless of the surgical or non-surgical group, the native T1 value of the recurrence subgroup was significantly higher than that of the non-recurrence subgroup (*P*<0.05). However, there was no significant difference between the ADC values of the recurrence and non-recurrence subgroups in both the surgical and non-surgical groups (*P*>0.05) (**Table 3**). The AUC of the native T1 value for the prediction of postoperative CC recurrence was 0.742. When native T1=1480.19 ms, its sensitivity and specificity were 76.9% and 70.3%, respectively. The AUC of the native T1 value for predicting the recurrence of CC after non-surgical treatment was 0.780. When native T1=1494.00 ms, its sensitivity and specificity were 80.0% and 75.0%, respectively (**Figure 4**).

**Table 2:** Chi-square test of clinical variables for predicting tumor recurrence.

Characteristics	Surgical group (n=77)		Non-surgical group (n=30)	
	χ^2^	*P* value	χ^2^	*P* value
FIGO stage	0.256	0.613	1.425	0.490
Histology type	0.428	0.513	1.667	0.197
Tumor differentiation	0.337	0.845	1.500	0.472
Maximum tumor size	0.929	0.335	2.400	0.121
Lymph node invasion	6.222	0.013	0.072	0.789

FIGO, International Federation of Gynecology and Obstetrics.

**Table 3:** Comparison of the native T_1_ values of the recurrence and non-recurrence subgroups after treatment.

Groups	Native T1 value (ms)	ADC value (×10^–3^mm^2^/s)
Surgical (n=77)		
Recurrence (n=13)	1516.77 ± 69.27	0.71 ± 0.11
Non-recurrence (n=64)	1433.55 ± 122.96	0.73 ± 0.12
t	2.357	–0.547
*P* value	0.021	0.586
Non-surgical (n=30)		
Recurrence (n=10)	1544.53 ± 104.24	0.75 ± 0.11
Non-recurrence (n=20)	1442.29 ± 114.42	0.77 ± 0.11
t	2.359	–0.372
*P* value	0.026	0.712

ADC, apparent diffusion coefficient.

**Figure 5:**
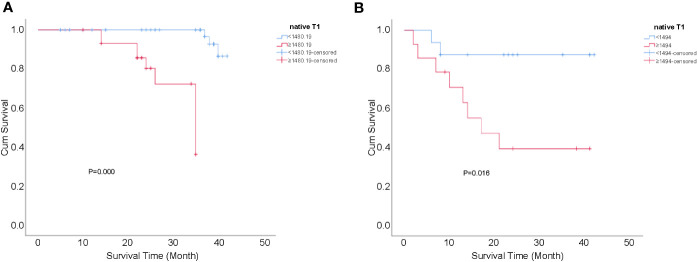
Kaplan-Meier curves for the cumulative recurrence-free survival rate of patients with CC. **(A)** native T1 values ≥1480.19 vs <1480.19 ms in the surgical group, *P*=0.000; and **(B)** native T1 values ≥1494.00 vs <1494.00 ms in the non-surgical group, *P*=0.016.

Finally, logistic regression analysis showed that associated risk factors included lymph node invasion and the native T1 value in the surgical group (*P*=0.003 and 0.004, respectively); meanwhile, only the native T1 value was a significant risk factor of recurrence in patients with CC after non-surgical treatment (*P*=0.040) (**Table 4**). Regardless of the surgical group or the non-surgical group, recurrence-free survival rates of CC with native T1 values higher than the optimal cut-offs (1480.19 and 1494.00 ms, respectively) were significantly lower compared with those with values lower than the optimal cut-offs (*P*=0.000 and 0.016, respectively) (**Figure 5**).

**Table 4:** Results of logistic regression analysis of clinical variables and native T_1_ value for the prediction of the recurrence of cervical cancer after treatment.

Index	Surgery		Non-surgery
	Lymph node invasion	Native T1 value	Native T1 value
B	2.606	0.011	0.009
SE	0.888	0.004	0.004
Wald	8.609	8.217	4.218
*P* value	0.003	0.004	0.040
OR (95% CI)	13.548 (2.376–77.261)	1.011 (1.003–1.019)	1.009 (1.000–1.018)

B, beta; SE, standard error; OR, odds ratio; CI, confidence interval.

## Discussion

Previous studies have demonstrated that FIGO stage, histology type, histology grade, tumor size, and lymph node invasion were important prognostic factors of CC, but these variables are insufficient to accurately predict clinical outcomes (7, 12, 14). In this study, we assessed the feasibility of T1 mapping to reflect the recurrence of CC. The results showed that lymph node invasion was significantly associated with tumor recurrence only in the surgical group (*P*<0.05). Moreover, as quantitative parameters of T1 mapping, native T1 values of tumors in the surgical and non-surgical groups could be used to effectively identify patients with a high risk of relapse after therapy (*P*<0.05). However, the ADC values of the two subgroups were not significantly different (*P*>0.05). The results also indicated that patients with higher native T1 values (≥cut-offs) tend to have higher incidences of CC recurrence.

As is well elucidated in the literature, lymph node metastasis plays an important role in determining the oncological prognosis and treatment method in patients with CC (34). Even in early-stage CC, Tsunoda et al. found that the incidence rate of lymph node metastasis ranges from 17–33% (35). Üreyen et al. reported on 27 early-stage CC patients with recurrence and found that lymph node invasion was significantly relevant to recurrence after surgical treatment (36). Another study by Mabuchi et al. revealed that it was the presence instead of the number and location of lymph node metastasis that independently affected the survival in patients with CC treated by salvage hysterectomy plus lymphadenectomy (37). Our result showed that the presence of metastatic lymph nodes was a significant risk factor for the recurrence of CC in the surgical group, which was in line with the previously mentioned reports. It is reported that more than 80% of metastatic lymph nodes are smaller than 10 mm and more than 50% are smaller than 5 mm (38). Hence, the size criterion for evaluating lymph node status by radiography has some limitations, potentially explaining why lymph node metastasis was not a significant risk factor in the non-surgical group.

Native T1 values represent critical physical parameters of MRI and are related to many factors, such as tissue water content, cell density, and macromolecular concentration (39). These values are particularly sensitive to alterations in water content and can distinguish microscopic changes in tissues that are not easily displayed on conventional T1WI (22). Olsen et al. (40) proposed that native T1 values significantly correlate with the Ki-67 index, a biomarker of tumor cell proliferation activity (41). Due to variations in tumor cell proliferation activities, water content varies between tissues, leading to differences in the corresponding native T1 values (27). Moreover, previous research on liver cancer has shown that recurrence is associated with tumor heterogeneity and type (42, 43). It has been reported that a decrease in tumor heterogeneity generally corresponds to improved outcomes (44). Ditmer et al. (45) used texture analysis to discriminate high- and low-grade gliomas quantitatively and proposed that tumor grade is strongly correlated with heterogeneity. Adams et al. (30) analyzed the native T1 values of patients with renal clear cell carcinoma and showed that native T1 values gradually increase with increasing grades. They also reported that there was a statistical difference between the low-level and high-level groups in their study (*P*<0.05). Thus, we speculated that the increased native T1 values of the patients in the recurrent subgroup in the present study might be related to increased tissue water content, active cell proliferation, and evident heterogeneity.

In addition, we found that there was no significant difference in ADC values between the recurrence and non-recurrence subgroups in the surgical and non-surgical groups. Although DWI has been widely used to predict tumor treatment outcomes, Somoye et al. (46) did not find any evidence of a relationship between survival in patients with CC and pre-treatment baseline ADCmean and suggested that it was insufficient for ADCmean to predict the prognosis of tumors. We speculated that the absence of significant difference might be due to the integrated effects of diffusion and microperfusion on ADC values calculated based on the Gaussian distribution model (15).

The present study's logistic regression analysis showed that the native T1 values helped identify patients with CC at high risk for recurrence. Furthermore, we calculated the cut-off value of native T1 using the Youden index in the ROC curve and analyzed the patients who received standardized treatment for CC. The results indicated that the optimal cutoff native T1 value for predicting the recurrence of CC after surgical and non-surgical treatment is 1480.19 ms and 1494.00 ms, respectively. According to Kaplan–Meier analysis, if the native T1 value of a patient who underwent surgery is ≥1480.19 ms, and that of a patient who received non-surgical treatment is ≥1494.00 ms, clinicians should be highly vigilant and strongly consider the possibility of recurrence; this will facilitate the timely adjustment of the subsequent treatment plan and time interval for the follow-up to reduce the risk of treatment failure and improve the quality of life and survival rates of patients with CC.

This study had some limitations. First, the study population was relatively small, especially the number of patients in the recurrence subgroup. Second, the ROI of lesions may contain small necrotic areas that are invisible to the naked eye, which may have affected the accuracy of measurements. Further studies on whole lesion texture analysis based on T1 mapping are needed to rectify any effect of selection bias on the results of the present study. Third, the follow-up duration was relatively short. Furthermore, only one scanner and a single T1 mapping sequence were used for image acquisition. In addition, reproducibility across different MRI devices and imaging protocols was not tested; thus, the results may not be generalizable. In the future, we will validate our findings and promote the clinical application of this technique using multicenter studies with larger patient cohorts and long-term follow-up periods.

Compared with ADC, the pre-treatment native T1 value is a significant risk factor for CC recurrence. Furthermore, risk assessment of recurrence using a noninvasive method will provide a rational basis for further improvement of therapeutics.

## Data availability statement

The original contributions presented in the study are included in the article/supplementary material. Further inquiries can be directed to the corresponding author.

## Ethics statement

The studies involving human participants were reviewed and approved by Ethics Committee of the First Affiliated Hospital of Zhengzhou University. The patients/participants provided their written informed consent to participate in this study. Written informed consent was obtained from the individual(s) for the publication of any potentially identifiable images or data included in this article.

## Author contributions

Guarantor of integrity of the entire study: JL and SL. Study concepts and design: JL and JC. Literature research: JL. Clinical studies: JL, SL, and QC. Experimental studies/data analysis: JL, SL, QC, YW, and JZ. Statistical analysis: JL. Manuscript preparation: JL. Manuscript editing: YZ, MN, YW, and JZ. All authors read and approved the final manuscript. All authors contributed to the article and approved the submitted version.
